# Expanding the spectrum of A20 haploinsufficiency in two Chinese families: cases report

**DOI:** 10.1186/s12881-019-0856-1

**Published:** 2019-07-12

**Authors:** Guo-min Li, Hai-mei Liu, Wan-zhen Guan, Hong Xu, Bing-bing Wu, Li Sun

**Affiliations:** 1 Department of Rheumatology, Children’s Hospital of Fudan University, 399 Wan-yuan road, Shanghai, 201102 China; 2 Medical Transformation Centre, Children’s Hospital of Fudan University, 399 Wan-yuan road, Shanghai, 201102 China

**Keywords:** A20 haploinsufficiency, Hypothyroidism, Interstitial lung disease, Liver fibrosis, Macrophage activation syndrome, *TNFAIP3* gene

## Abstract

**Background:**

The association between mutations in the TNFAIP3 gene and a new autoinflammatory disease (called A20 haploinsufficiency, HA20) has recently been recognized. Here, we describe four patients with HA20 from two unrelated Chinese families.

**Case presentation:**

A total of four patients from two families were included. The average age at onset was 5.9 years. All patients had no signs of eye or skin problems, such as uveitis, rash, folliculitis and dermal abscess. Prior to the recognition of HA20, P1 was diagnosed with SLE, liver fibrosis and hypothyroidism. She also had no oral, genital or perineal ulcers. P2 was diagnosed with Crohn’s disease and inflammatory bowel disease-related arthritis (IBD-RA). He had a perianal abscess but no oral or genital ulcers. P3, the father of P1 and P2, only had mild oral ulcers, arthralgia, and archosyrinx. P4 was diagnosed with polyarticular juvenile idiopathic arthritis (JIA), macrophage activation syndrome (MAS) and interstitial lung disease (ILD). Whole exome sequencing (WES) was performed in two families. WES revealed heterozygous c.559C > T in the TNFAIP3 gene in P1, P2 and P3, while the c.259C > T mutation in the TNFAIP3 gene was identified in P4. The c.259C > T mutations is novel.

**Conclusion:**

HA20 had a different phenotype between families and even between family members with the same mutation. Liver fibrosis, hypothyroidism, ILD and MAS in the patients with HA20 were first reported in this study. Our results expanded the phenotype and genotype spectrum of A20 haploinsufficiency.

**Electronic supplementary material:**

The online version of this article (10.1186/s12881-019-0856-1) contains supplementary material, which is available to authorized users.

## Background

The *TNFAIP3* gene that encodes the 90 kDa A20 protein was first described in 1990 as a TNF-α-induced primary response gene in endothelial cells [1]. The protein A20 is a negative regulator of the TNF-nuclear factor-kB signaling pathway, which plays fundamental roles in various physiological and pathological processes, such as immunity, apoptosis, inflammation, and cancer [2, 3]. Polymorphisms in *TNFAIP3* have been linked to the development of several autoimmune and autoinflammatory diseases in genome-wide association studies, such as systemic lupus erythematosus [4–6], Sjögren’s Syndrome [7], Crohn disease [8], rheumatoid arthritis [9, 10], type 1 diabetes [11] and psoriasis [12]. Recently, heterozygous germline mutations in the *TNFAIP3* gene have been found to cause the haploinsufficiency of A20 (HA20), which displays an early-onset autoinflammatory disease resembling Behçet’s disease [13]. HA20, first described by Zhou et al. in 2016, is a new autoinflammatory disease [13]. Zhou et al. found that the major phenotype of HA20 displays Behçet’s disease-like symptoms, including recurrent aphthous stomatitis, genital ulcers, and intestinal symptoms [13]. However, a number of studies showed that some patients present with not only the features of autoinflammatory diseases but also several autoimmune-like features [14–17]. Therefore, these preceding clinical reports suggested that there might be unexpected phenotypes in HA20. Cases with HA20 were not reported in China. In this study, we described four patients in two HA20 families, which were diagnosed by whole exons sequencing, to enrich the phenotype and genotype spectrum of HA20.

## Case presentation

**Fig. 1:**
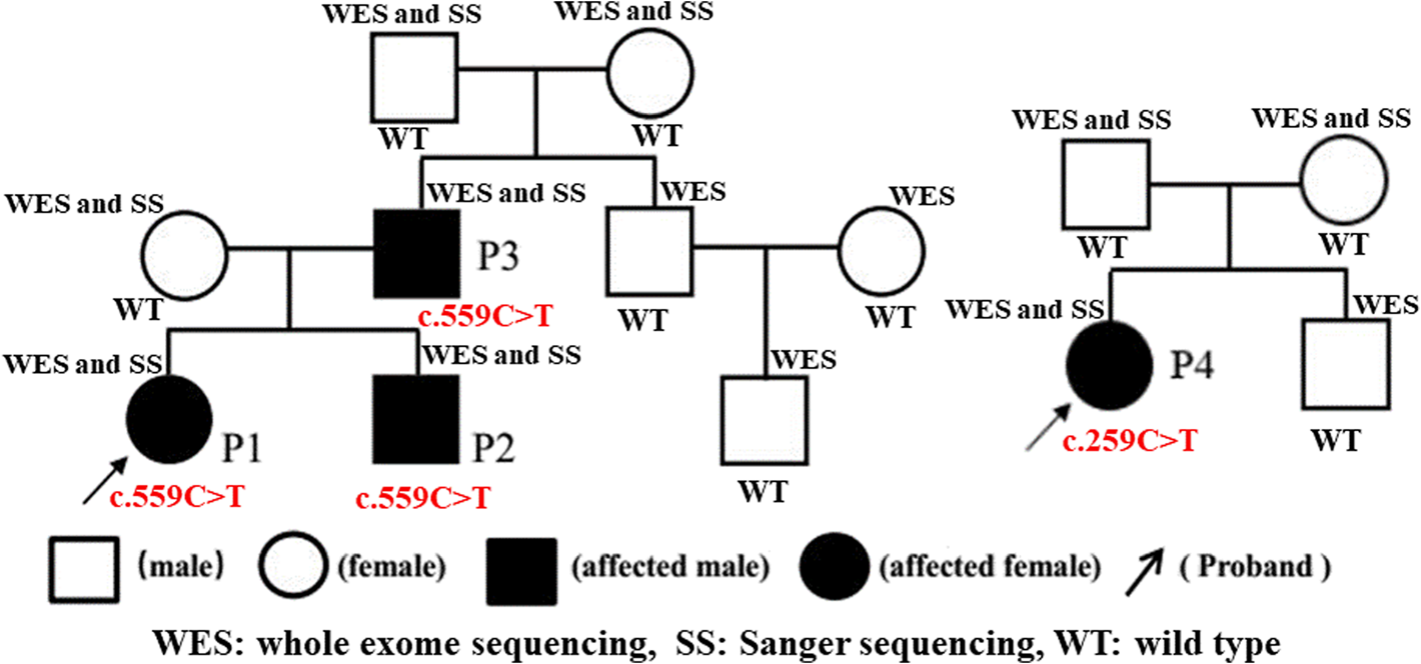
Spectrum of family 1 and family 2

Two unrelated families with HA20 were enrolled in this study (Fig. 1). All adults provided written informed consent.

### Family 1

**Fig. 2:**
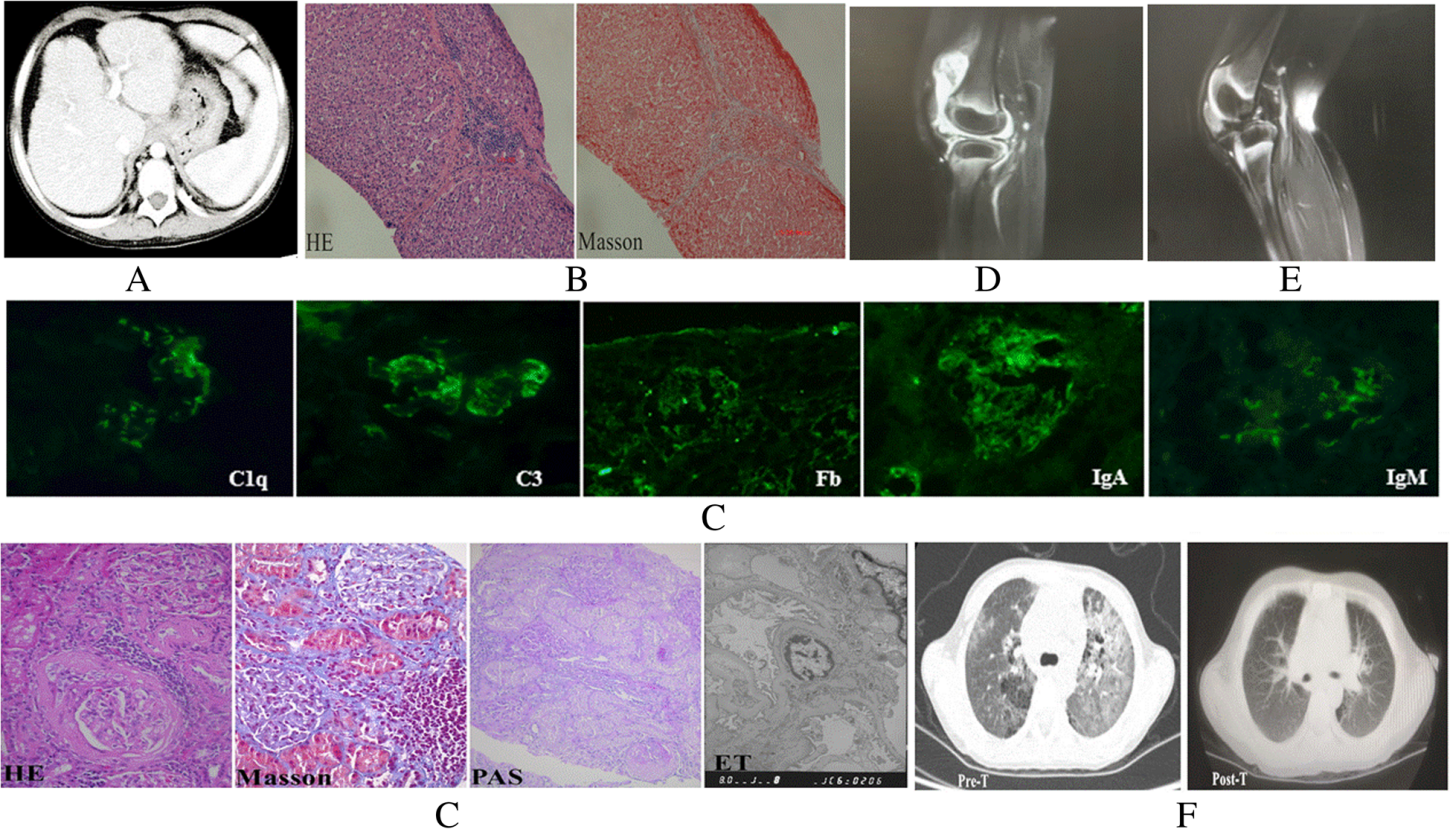
**a** Abdominal contrast-enhanced MRI revealed hepatomegaly and hepatic fibrosis in P1. **b** Liver biopsy showed hepatic fibrosis by HE and Masson stain (× 200) in P1. **c** Renal biopsy showed lupus nephritis type III under light (× 400), electron (× 11600) and Immunofluorescence (× 400) microscopy in P1. **d** Contrast-enhanced MRI revealed chronic synovitis of the knee joint in P2. **e** Contrast-enhanced MRI revealed chronic synovitis of the knee joint in P3. **f** Lung CT scan showed interstitial lung disease (ILD) in P3

The proband 1 (P1), a 14-year-old Chinese girl, presented with abdominal swelling at the age of 7. She went to her local hospital, and ultrasound showed hepatomegaly, ascites and pericardial effusion. Laboratory findings suggested dysfunction of the liver and thyroid. Therefore, hepatic fibrosis, pericardial effusion and hypothyroidism were diagnosed. She was referred to our hospital for evaluation because of the continued hepatomegaly with hepatic dysfunction for 4 years and intermittent fever for 6 months at the age of 11. Laboratory testing revealed leukopenia, anemia, thrombocytopenia, hematuria, proteinuria, low levels of complement, and high levels of erythrocyte sedimentation rate and C-reactive protein. Coombs test was positive. ANA and SSA were positive, while other autoantibodies were negative. Abdominal contrast-enhanced MRI revealed hepatomegaly and hepatic fibrosis (Fig. 2a). She was diagnosed with hypothyroidism, hepatic fibrosis, systemic lupus erythematosus and lupus nephritis. Liver biopsy was performed due to continued hepatomegaly and hepatic dysfunction and showed liver fibrosis (Fig. 2b). Renal biopsy was also done because of persistent hematuria and proteinuria and displayed moderately increased mesangial matrix and mesangial hypercellularity under the light microscope; subepithelial deposits were noted, and some mesangial changes were possibly present by electron microscopy. Immunofluorescence was positive forC1q, C3, IgA, IgM, and Fb (Fig. 2c). Oral prednisolone and hydroxychloroquine (HCQ) combined with mycophenolate mofetil (MMF) were given to her*.* Six months later, the level of complement was restored to normal, hematuria and proteinuria disappeared, and liver function returned to normal. The patient is currently receiving anti-TNFα (etanercept 25 mg/week) in combination with HCQ, low dose prednisolone and MMF. The treatment has displayed good efficacy.

Patient 2 (P2), the 5.1-year-old younger brother of P1, presented with a fever of unknown origin and had polyarthritis at age 2.6. He was initially diagnosed with polyarticular juvenile idiopathic arthritis (JIA) because of the chronic synovitis of his joints, which was found in a local hospital (Fig. 2d). He was admitted to our hospital for further assessment due to abdominal pain, diarrhea and perianal abscess after discharge from the local hospital. On admission, HLA-B27 was positive, but no significant autoantibody was detected in his serum. Under endoscopy, multiple ulcers were found on the gastrointestinal tract. The change of pathology was consistent with Crohn’s disease. Therefore, the patient was diagnosed with Crohn’s disease and inflammatory bowel disease-related arthritis (IBD-RA). Prednisolone, Etiasa and MTX were given to him, and his joint symptoms and intestinal symptoms disappeared gradually. He is currently being treated with anti-TNFα (etanercept 12.5 mg/ 5 days), Etiasa and MTX. The treatment has displayed good efficacy.

Patient 3 (P3), the father of P1 and P2, also had a history of recurrent arthralgia and anal fistula starting from 7 years of age. He refused further assessment.

### Family 2

Patient 4 (P4), a 9-year-old girl, visited a local hospital complaining of a fever of unknown origin and polyarthritis at the age of 7 years. She was also diagnosed with polyarticular juvenile idiopathic arthritis (JIA) because of the chronic synovitis of her joints (Fig. 2e). She was referred to our hospital for evaluation due to oral ulcers, cough, and positive ANA and anti-ds DNA antibodies after discharge from the local hospital. She had no relevant past medical history and no family history of rheumatic diseases. On admission, laboratory studies showed hyperferritinemia (> 2000 ng/ml), hypertriglyceridemia (5.57 mmol/L), hypofibrinogenemia (2.0 g/L), and increased levels of Alanine Aminotransferase (61 IU/L), Glutamic-oxalacetic Transaminase (97 IU/L) and lactate dehydrogenase (726 IU/L). HLA-B27 and ANA were positive for her, but no other autoantibody was detected in her serum. Lung CT scan showed interstitial lung disease (ILD) (Fig. 2f). Therefore, she was diagnosed with JIA, macrophage activation syndrome (MAS) and ILD. Voltaren, prednisolone, and CSA were given to her, and her temperature became stable while joint symptoms disappeared quickly. She is currently receiving anti-TNFα (etanercept 25 mg/week) in association with low dose prednisolone. The treatment has displayed good efficacy, and ILD was significantly improved (Fig. 2f).

The demographic and clinical features of the patients are summarized in Table 1 and Table 2, respectively. The laboratory findings of pre-therapy and post-therapy in the patients are summarized in Table 3.

**Table 1:** Characteristics of patients with HA20

Patient no	Family	Gender	Current age	Age at onset	Previous diagnosis	Current diagnosis	Previous treatment	Current treatment
1	Family 1	F	14.1 years	7.0 years	liver fibrosis Hypothyroidism SLE Lupus nephritis	HA20	Prednisolone MMF Sodium levothyroxine HCQ	Prednisolone MMF Sodium levothyroxine HCQ Etanercept
2	Family 1	M	5.1 years	2.5 years	Crohn’s disease IBD-RA	HA20	Prednisolone 5-AminoSalicylicAcid MTX	Prednisolone 5-AminoSalicylicAcid MTX Etanercept
3	Family 1	M	38.2 years	7.0 years	–	HA20		
4	Family 2	F	8.3 years	6.9 years	JIA, CTD-ILD MAS	HA20	Prednisolone CSA Diclofenac Sodium	Prednisolone CSA Diclofenac Sodium Etanercept

*SLE* Systemic lupus erythematosus, *IBD-RA* Inflammatory bowel disease-related arthritis, *CTD-ILD* Connective tissue disease-related interstitial lung disease, *MAS* Macrophage activation syndrome, *HCQ* Hydroxychloroquine, *MTX* Methotrexate, *CSA* Cyclosporine A

**Table 2:** Clinical features of patients with *TNFAIP3* mutation

No	Fever	Ulcers	Musculoskeletal	Cardiovascular	Intestinal	Thyroid	Liver	lung	Genotype	Mutation origin
1	Yes	No	No	pericardial effusion	No	Hypothyroidism	Hepatomegaly liver fibrosis	No	c.559C > T (p.Q187X)	Father
2	Yes	No	Polyarthritis	No	Diarrhea perianal abscess Crohn’s disease	No	No	No	c.559C > T (p.Q187X)	Father
3	No	Oral	Arthralgia	No	Archosyrinx	No	No	No	c.559C > T (p.Q187X)	De novo
4	Yes	No	Polyarthritis	No	No	No	No	Cough ILD	c.259C > T (p.R87X)	De novo

*ILD* Interstitial lung disease

**Table 3:** Laboratory findings of patients with HA20

Testing	Case 1		Case 2		Case 4		Normal range
	Pretherapy	posttherapy	Pretherapy	posttherapy	Pretherapy	posttherapy	
ESR (mm/h)	89.0	19	35	11	79.0	9.0	0–20
CRP (mg/dl)	48.0	< 8	89	< 8	48.0	< 8	< 8
Hemoglobin (g/dl)	89.2	115.0	85.2	129.0	117	142	110–160
Leukocyte (/mm3)	3.5	6.8	11.2	13.9	13.5	13.5	4–10
Thrombocyte (/mm3)	81.0	118.0	205	304	528	238	100–300
TNF-α(pg/ml)	22.1	4.2					0.0–8.1
IL-6	46.62	8.62	49.6	5.77	31.51	6.21	< 7
Ferritin	111.6	33.8	447.0	31.0	> 2000	237.3	0.94–71.7
C3(g/L)	0.22	0.66	1.65	1.58	1.87	1.42	0.67–1.76
C4(g/L)	0.06	0.1	0.56	0.72	0.3	0.36	0.1–0.4
CH50(U/ml)	10.0	26	66	58	35	26	23–46
ANA	+	+	–	–	+	–	Negative
Anti-dsDNA	+	–	–	–	–	–	Negative

### Whole exome sequencing

Whole exome sequencing (WES) was performed in two HA20 families, which included all members in Fig. 1. Methodology of WES and Sanger sequencing refers to our published work [18]. An average of 11.8Gb of raw sequence data was generated with 92.65× depth of exome target regions for each individual as paired-end 150 base pair reads. 91.2% of the raw date sequencing quality was above Q30. The coverage of at least 10× and 20× of the target regions was 99.52 and 97.5% respectively.

WES revealed heterozygous c.559C > T(p.Q187X) mutation in the TNFAIP3 gene (RS:NM_006290) in P1, P2 and P3, while the mutation was not detected in other members in family 1. The c.259C > T (p.R87X) mutation in the TNFAIP3 gene (RS:NM_006290) was identified in P4, but the mutation was not found in her parents.

Therefore, the c.559C > T and c.259C > T mutations were de novo for P3 and P4, respectively. No mutations were detected in other genes associated with autoinflammatory diseases (see the Additional file 1) in two families.

### Sanger sequencing

**Fig. 3:**
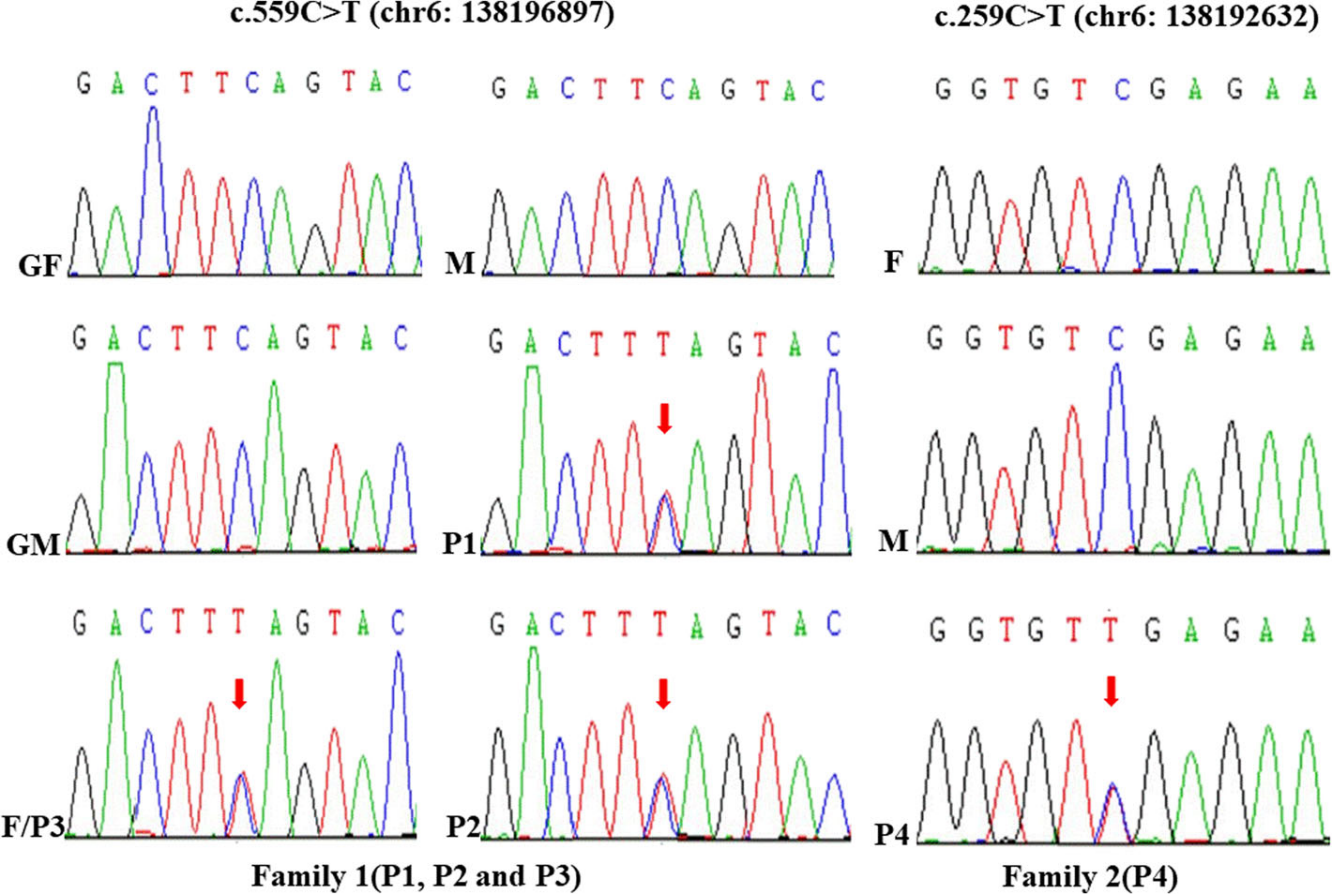
Mutation analysis in *TNFAIP3* gene in family 1 and family 2. P: patient; F: father; M: mother; GF: grandfather; GM: grandfather

Sanger sequencing was used to confirm mutations identified by WES. Methodology of Sanger sequencing also refers to our published work [18]. Two mutations, c.559C > T and c.259C > T in the TNFAIP3 gene, were confirmed by Sanger sequencing in two families (Fig. 3). They were checked in mutation databases on human populations, such as ExAC Browser (http://exac.broadinstitute.org/), 1000Genomes (http://www.internationalgenome.org/), and HGMD (http://www.hgmd.cf.ac.uk/ac/index.php). The c.259C > T mutation was not found in the above mutation databases.

## Discussion and conclusion

In this study, we describe the clinical manifestations of four Chinese patients with HA20 from two unrelated families. Four patients all had no signs of eye and skin problems, such as uveitis, rash, folliculitis and dermal abscess. Based on clinical features, P1 was initially diagnosed with SLE, liver fibrosis and hypothyroidism. She also had no oral, genital or perineal ulcers. P2 was initially diagnosed with Crohn’s disease and inflammatory bowel disease-related arthritis (IBD-RA). He had perianal abscess but no oral or genital ulcers. However, P3, the father of P1 and P2, only had mild oral ulcers, arthralgia, and archosyrinx. Therefore, three patients had different phenotypes in this family. Furthermore, signs of liver and thyroid problems were never reported before in HA20 patients in other studies.

P4 was initially diagnosed with polyarticular juvenile idiopathic arthritis (JIA) because of the chronic synovitis of her joints. Although ANA was positive, no other autoantibody was detected in her serum. She did not meet the classification criteria for SLE. However, she met the classification criteria of macrophage activation syndrome (MAS) in suspected systemic juvenile idiopathic arthritis (SJIA) due to fever, hyperferritinemia, hypertriglyceridemia and hypofibrinogenemia. She had a cough, and her lung CT scan showed ILD. ILD is one of the most common and clinically important manifestations of autoimmune diseases/connective tissue disease [19]. To date, MAS and ILD in the HA20 patients from this study were the first cases reported. Thus, clinical features were significantly different between families with HA20 in our study.

HA20 was initially identified as a Behçet’s disease-like phenotype. The original description showed that all patients with HA20 had recurrent aphthous stomatitis and genital ulcers [13]. However, not all patients included in our study and other studies developed some Behçet’s disease-like phenotypes [14–17], even if some patients included in our study and other studies did not have a Behçet’s disease-like phenotype [15–17]. Thus, HA20 is a very heterogeneous disease. Patients presenting with Behçet’s-like disease or other features of autoinflammatory and autoimmune disease starting in early childhood should be screened for the *TNFAIP3* mutation, especially if there is a family history of similar symptoms, as the clinical course and response to the treatment of this genetic disorder differs from common Behçet’s disease.

A20 functions as a negative regulator of the nuclear factor kB (NF-kB) pathway and modulates immune responses by preventing excessive activation of NF-kB in response to a variety of external stimuli [20]. It is structurally divided into two types of domains – the N-terminal Ovarian tumor (OTU) domain and the C-terminal domain containing 7 zinc finger motifs (ZnF1–ZnF7) [21]. It harbors a deubiquitination enzyme domain and activates multiple mechanisms to antagonize ubiquitination upstream of the NF-kB essential modulator (NEMO), a regulatory subunit of the IkB kinase (IKK) complex [4]. The IKK complex has 2 catalytic subunits (IKKa and IKKb) and a subunit that regulates the canonical NF-kB pathway (NEMO/IKKg). The IKK regulates the noncanonical pathway by IKKa [22]. The ubiquitin-induced recruitment of A20 to NEMO can downregulate IKK activation by blocking IKK phosphorylation. On activation, IKK phosphorylates the inhibitory IkBa protein, leading to its degradation and dissociation from NF-kB, after which it translocates to the nucleus [23]. Hence, A20 deficiency leads to the activation of the NF-kB pathway. Both a mutation in the *TNFAIP3* gene in the N-terminal domain or the C-terminal domain can cause the loss of A20 function [14–17]. Approximately 10 different disease-causing mutations of the *TNFAIP3* gene have been reported in various populations (http://www.hgmd.cf.ac.uk/, last updated April, 2018). In our study, WES revealed that a heterozygous c.559C > T(p.Q187X) mutation in the *TNFAIP3* gene cosegregates with the disease in three patients of family 1. The mutation was confirmed by Sanger sequencing, but it was reported by other study [16]. By sequence analysis in family 1, we found that P3 had this de novo mutation and transmitted it to his children. Interestingly, the three patients had the same genotype of the *TNFAIP3* gene but had different phenotypes. The heterozygous c.259C > T (p.R87X) mutation in the *TNFAIP3* gene was detected by WES and confirmed by Sanger sequencing in family 2. Sequence analysis in family 2 showed that the p.R87X mutation is a de novo mutation, where a nonsense mutation leads to the truncation of A20. The p.R87X mutation was first reported in our study. The p.Q187X and p.R87X mutations are both located in OUT domains, which controlled NF-κB signaling by deubiquitinating receptor-interacting protein (RIP) 1, RIP2 and TNF receptor-associated factor (TRAF) [24].

Here, we provide four HA20 patients with novel features of considerable existing evidence implicating novel mutations in the *TNFAIP3* gene. Our study expands the phenotype and genotype spectrum of HA20.

HA20 is a new autoinflammatory disease caused by heterozygous loss-of-function mutations in *TNFAIP3*. These mutations cause insufficient DUB activity of A20 and lead to an increased NF-κB signaling and phosphorylation of c-Jun N-terminal kinase and p38 mitogen-activated protein kinases (MAPKs). Clinical features were significantly different between families with HA20, even between numbers with the same *TNFAIP3* mutation.

## Additional file

Additional file 1: Genes associated with auto-inflammatory diseases. (DOCX 18 kb)

## Data Availability

All data generated or analyzed during this study are included in this published article.

## Abbreviations

ANA: Antinuclear antibody
CSA: Cyclosporine A
HA20: A20 haploinsufficiency
HCQ: Hydroxychloroquine
IBD-RA: Inflammatory bowel disease-related arthritis
IKK: The IkB kinase
ILD: Interstitial lung disease
JIA: Juvenile idiopathic arthritis
MAPKs: Mitogen-activated protein kinases
MAS: Macrophage activation syndrome
MMF: Mycophenolate mofetil
NEMO: NF-kB essential modulator
NF-kB: The nuclear factor kB
OUT: The N-terminal Ovarian tumor
SJIA: Systemic juvenile idiopathic arthritis
SSA: Sjogren’s syndrome A antigen
WES: Whole exome sequencing
