# Supplementary material for: Expanding the spectrum of A20 haploinsufficiency in two Chinese families: cases report
Source: BMC Med Genet. 2019 Jul 12;20:124. doi: 10.1186/s12881-019-0856-1 (PMC6624950; doi:10.1186/s12881-019-0856-1)
Supplement: Supplementary file 1 — Genes associated with auto-inflammatory diseases. (DOCX 18 kb) [file 12881_2019_856_MOESM1_ESM.docx]

**Genes associated with auto-inflammatory diseases**

| Gene | Interpretation of Gene Names | OMIM | Locus | Transmission | Disease |
| --- | --- | --- | --- | --- | --- |
| **AP1S3** | **Adaptor-related protein complex 1, sigma-3 subunit** | [**615781**](http://omim.org/entry/615781) | [**2q36.1**](http://omim.org/geneMap/2/830?start=-3&limit=10&highlight=830) | Dominant | Angioedema, hereditary, type I |
| ***C1NH*** | **Complement Component 1 Inhibitor** | [**606860**](http://omim.org/entry/606860) | [**11q12.1**](http://genome.ucsc.edu/cgi-bin/hgTracks?db=hg19&hgFind=omimGeneAcc&position=606860) | **Dominant** | **Psoriasis susceptibility 2** |
| ***CARD14*** | **Caspase recruitment domain-containing protein 14** | [**607211**](http://omim.org/entry/607211) | [**17q25.3**](http://genome.ucsc.edu/cgi-bin/hgTracks?db=hg19&hgFind=omimGeneAcc&position=607211) | **Dominant** | **Psoriasis susceptibility 2** |
| ***CARD14*** | **Caspase recruitment domain-containing protein 14** | [**607211**](http://omim.org/entry/607211) | [**17q25.3**](http://genome.ucsc.edu/cgi-bin/hgTracks?db=hg19&hgFind=omimGeneAcc&position=607211) | **Dominant** | **Pityriasis rubra pilaris** |
| ***CECR1*** | **Cat eye syndrome chromosome region, candidate 1** | [**607575**](http://omim.org/entry/607575) | [**22q11.2**](http://genome.ucsc.edu/cgi-bin/hgTracks?db=hg19&hgFind=omimGeneAcc&position=607575) | **Recessive** | **Polyarteritis nodosa, childhood-onset** |
| ***IL10*** | **Interleukin 10** | [**124092**](http://omim.org/entry/124092) | [**1q32.1**](http://genome.ucsc.edu/cgi-bin/hgTracks?db=hg19&hgFind=omimGeneAcc&position=124092) | **Recessive** |  |
| ***IL10RA*** | **Interleukin 10 receptor, alpha** | [**146933**](http://omim.org/entry/146933) | [**11q23.3**](http://genome.ucsc.edu/cgi-bin/hgTracks?db=hg19&hgFind=omimGeneAcc&position=146933) | **Recessive** | **Inflammatory bowel disease 28** |
| ***IL10RB*** | **Interleukin 10 receptor, beta** | [**123889**](http://omim.org/entry/123889) | [**21q22.11**](http://genome.ucsc.edu/cgi-bin/hgTracks?db=hg19&hgFind=omimGeneAcc&position=123889) | **Recessive** | Inflammatory bowel disease 25 |
| ***IL1RN*** | **Interleukin 1 receptor antagonist** | [**147679**](http://omim.org/entry/147679) | [**2q13**](http://genome.ucsc.edu/cgi-bin/hgTracks?db=hg19&hgFind=omimGeneAcc&position=147679) | **Recessive** | Osteomyelitis, Sterile Multifocal, With Periostitis And Pustulosis |
| ***IL36RN*** | **Interleukin 36 receptor antagonist** | [**605507**](http://omim.org/entry/605507) | [**2q13**](http://genome.ucsc.edu/cgi-bin/hgTracks?db=hg19&hgFind=omimGeneAcc&position=605507) | **Recessive** | **Pustular Psoriasis, Generalized** |
| ***LPIN2*** | **LIPIN 2** | [**605519**](http://omim.org/entry/605519) | [**18p11.31**](http://genome.ucsc.edu/cgi-bin/hgTracks?db=hg19&hgFind=omimGeneAcc&position=605519) | **Recessive** | **Majeed syndrome** |
| ***MEFV*** | **MEditerranean FeVer** | [**608107**](http://omim.org/entry/608107) | [**16p13**](http://genome.ucsc.edu/cgi-bin/hgTracks?db=hg19&hgFind=omimGeneAcc&position=608107) | **Recessive** | **Familial Mediterranean Fever** |
| ***MVK*** | **Mevalonate Kinase** | [**251170**](http://omim.org/entry/251170) | [**12q24**](http://genome.ucsc.edu/cgi-bin/hgTracks?db=hg19&hgFind=omimGeneAcc&position=251170) | **Recessive** | **Hyper IgD Syndrome** |
| ***MVK*** | **Mevalonate Kinase** | [**251170**](http://omim.org/entry/251170) | [**12q24**](http://genome.ucsc.edu/cgi-bin/hgTracks?db=hg19&hgFind=omimGeneAcc&position=251170) | **Recessive** | **Mevalonic Aciduria** |
| ***MVK*** | **Mevalonate Kinase** | [**251170**](http://omim.org/entry/251170) | [**12q24**](http://genome.ucsc.edu/cgi-bin/hgTracks?db=hg19&hgFind=omimGeneAcc&position=251170) | **Dominant** | **Porokeratosis 3, Disseminated Superficial Actinic Type** |
| ***NLRC4*** | **NLR family, caspase recruitment domain-containing 4** | [**606831**](http://omim.org/entry/606831) | [**2p22.3**](http://omim.org/geneMap/2/133?start=-3&limit=10&highlight=133) | **Dominant** | **Autoinflammation with infantile enterocolitis** |
| ***NLRP12*** | **NLR pyrin domain containing protein 12** | [**609648**](http://omim.org/entry/609648) | [**19q13.42**](http://genome.ucsc.edu/cgi-bin/hgTracks?db=hg19&hgFind=omimGeneAcc&position=609648) | **Dominant** | **Familial Cold Autoinflammatory Syndrome 2** |
| ***NLRP1*** | **NLR pyrin domain containing protein 1** | [**606636**](https://omim.org/entry/606636) | [**17p13.2**](https://genome.ucsc.edu/cgi-bin/hgTracks?db=hg38&lastVirtModeType=default&lastVirtModeExtraState=&virtModeType=default&virtMode=0&nonVirtPosition=&position=chr17%3A5514754%2D5584512&hgsid=660327891_AMrWLMFW5ZK9nPC7MEzZwcntkNpF) | **Dominant/**  **Recessive** | Autoinflammation with arthritis and dyskeratosis |
| ***NLRP3*** | **NLR pyrin domain containing protein 3** | [**606416**](http://omim.org/entry/606416) | [**1q44**](http://genome.ucsc.edu/cgi-bin/hgTracks?db=hg19&hgFind=omimGeneAcc&position=606416) | **Dominant** | **Familial Cold Autoinflammatory Syndrome 1** |
| ***NLRP3*** | **NLR pyrin domain containing protein 3** | [**606416**](http://omim.org/entry/606416) | [**1q44**](http://genome.ucsc.edu/cgi-bin/hgTracks?db=hg19&hgFind=omimGeneAcc&position=606416) | **Dominant** | Muckle-Wells Syndrome |
| ***NLRP3*** | **NLR pyrin domain containing protein 3** | [**606416**](http://omim.org/entry/606416) | [**1q44**](http://genome.ucsc.edu/cgi-bin/hgTracks?db=hg19&hgFind=omimGeneAcc&position=606416) | **Dominant** | **Chronic Infantile Neurological Cutaneous and Articular Syndrome** |
| ***NLRP7*** | **NLR pyrin domain containing protein 7** | [**609661**](http://omim.org/entry/609661) | [**19q13.42**](http://genome.ucsc.edu/cgi-bin/hgTracks?db=hg19&hgFind=omimGeneAcc&position=609661) | **Recessive** | Hydatidiform Mole, Recurrent, 1 |
| ***NOD2*** | **Nucleotide-binding oligomerization domain 2** | [**605956**](http://omim.org/entry/605956) | [**16q12**](http://genome.ucsc.edu/cgi-bin/hgTracks?db=hg19&hgFind=omimGeneAcc&position=605956) | **Dominant** | **BLAU syndrome** |
| ***NOD2*** | **Nucleotide-binding oligomerization domain 2** | [**605956**](http://omim.org/entry/605956) | [**16q12**](http://genome.ucsc.edu/cgi-bin/hgTracks?db=hg19&hgFind=omimGeneAcc&position=605956) | **Dominant** | Early onset sarcoidosis |
| ***NOD2*** | **Nucleotide-binding oligomerization domain 2** | [**605956**](http://omim.org/entry/605956) | [**16q12**](http://genome.ucsc.edu/cgi-bin/hgTracks?db=hg19&hgFind=omimGeneAcc&position=605956) | **Dominant** | **Inflammatory bowel disease 1** |
